# Utility of Elevated Pentraxin-3 Level as Inflammatory Marker for Predicting Adverse Outcomes in Patients With Acute Coronary Syndrome: A Meta-Analysis

**DOI:** 10.3389/fcvm.2021.736868

**Published:** 2022-01-20

**Authors:** Yu Fan, Rong He, Changfeng Man, Dandan Gong

**Affiliations:** Institute of Molecular Biology and Translational Medicine, The Affiliated People's Hospital, Jiangsu University, Zhenjiang, China

**Keywords:** pentraxin-3, acute coronary syndrome, mortality, cardiac events, meta-analysis

## Abstract

**Background:**

Vascular inflammation plays an important role in the pathogenesis and development of acute coronary syndrome (ACS). However, studies on the association between elevated pentraxin-3 level and adverse outcomes in patients with ACS have yielded controversial results. The purpose of this meta-analysis was to assess the value of elevated pentraxin-3 level as an inflammatory marker for predicting adverse outcomes in patients with ACS.

**Methods:**

Two authors systematically searched the articles indexed in PubMed, Embase, CNKI, Wanfang, and VIP databases up to March 31, 2021. Studies reporting the association of elevated pentraxin-3 level at the acute phase with cardiovascular mortality, all-cause mortality, or cardiac events (cardiac death, non-fatal myocardial infarction, revascularization, or heart failure) in patients with ACS were included.

**Results:**

A total of 8,775 ACS patients from 12 studies were identified and analyzed. When compared the lowest pentraxin-3 level, ACS patients with the highest pentraxin-3 level conferred an increased risk of cardiovascular mortality [risk ratio (RR) 2.10; 95% CI 1.44–3.06], all-cause mortality (RR 1.99; 95% CI 1.46–2.71), and cardiac events (RR 1.74; 95% CI 1.32–2.29), even after adjustment for some important confounders. Subgroup analysis indicated that the association of elevated pentraxin-3 level with cardiac events appeared to be stronger in ST-segment elevation myocardial infarction patients (RR 2.72; 95% CI 1.69–4.36) than in all patients with ACS (RR 1.59; 95% CI 1.10–2.29).

**Conclusions:**

Elevated pentraxin-3 level is possibly an independent predictor of adverse outcomes in patients with ACS. Assessment of pentraxin-3 level at the acute phase can provide important information for early risk stratification of ACS.

## Introduction

Acute coronary syndrome (ACS) refers to a group of conditions, namely, ST-segment elevation myocardial infarction (STEMI), non-ST-segment elevation myocardial infarction (NSTEMI), and unstable angina. Despite advances in evidence-based therapies, ACS remains a major cause of morbidity and mortality around the world (1). However, risk prediction of morbidity and mortality is still challenging for patients with ACS.

The common cause of ACS is atherosclerotic plaque rupture or erosion with inflammation and subsequent coronary thrombosis (2). Increased local and systemic inflammation plays an important role in the development of ACS (3). Many inflammatory markers have been identified as strong and independent predictors of cardiovascular events in patients with ACS (4). Pentraxin-3 is mainly synthesized by macrophages, neutrophils, endothelial cells, and other cell types in response to acute inflammatory stimuli (5, 6). According to the primary structure of the subunit, the pentraxin family is divided into short pentraxins and long pentraxins (7, 8). Although from the same protein family as C-reactive protein (CRP), pentraxin-3 is predominantly expressed in atherosclerotic plaques (9). Therefore, pentraxin-3 may be a promising biomarker for inflammatory vascular disease. Apart from its diagnostic value (10, 11), several studies (12–19) have examined the role of pentraxin-3 level as a predictor of adverse outcomes in patients with ACS. However, the utility of pentraxin-3 as a prognostic biomarker remained conflicting in this population (20–22).

A previous meta-analysis has assessed the predictive value of elevated pentraxin-3 level in patients with coronary artery disease (CAD) (23). However, the results of this meta-analysis were limited by a small number of studies in the ACS subgroup. To clarify the pentraxin-3 as a prognostic biomarker, we undertook this meta-analysis to evaluate the value of elevated pentraxin-3 level for predicting adverse outcomes in ACS patients.

## Methods

### Data Sources and Searches

This meta-analysis was performed in accordance with the checklists of the Preferred Reporting Items for Systematic Reviews and Meta-analyses Statement (24). Two reviewers independently searched the articles indexed in PubMed, Embase, CNKI, Wanfang, and VIP databases up to March 31, 2021, using the following keywords in combination: “pentraxin-3” AND “acute coronary syndrome” OR “acute myocardial infarction” OR “unstable angina.” Reference lists of pertinent articles were manually reviewed to identify additional eligible studies.

### Study Selection

The inclusion criteria were as follows: (1) participants with clinical diagnosis of ACS (including STEMI, NSTEMI, and unstable angina); (2) cohort studies and *post hoc* analysis of clinical trials; (3) acute phase pentraxin-3 level as a predictor; (4) cardiovascular mortality, all-cause mortality, or cardiac events (cardiac death, recurrent myocardial infarction, revascularization, unstable angina pectoris, or heart failure) as an outcome of interests; and (5) reported multivariate-adjusted risk ratio (RR), hazard ratio (HR) or odds ratio (OR) and corresponding 95% CI of adverse outcomes for the highest pentraxin-3 group vs. the lowest pentraxin-3 group. The non-inclusion criteria included: (1) participants were not restricted in patients with ACS (enrolling all types of CAD); (2) risk estimate reported by univariate analysis; (3) analyzed the predictive value of pentraxin-3 level by continuous data; and (4) reviews or conference abstract.

### Data Extraction and Quality Assessment

Two reviewers independently scanned the titles or abstracts, retrieved the potentially eligible full-text articles, and evaluated the methodological quality. Any disagreements were resolved by discussion with a third reviewer. The extracted data included: a surname of the first author, time of publication, the origin of patients, study design, type or subtype of patients, sample sizes, age, gender distribution, the definition of cardiac events, cutoff value of the elevated pentraxin-3 level, length of follow-up, maximally adjusted risk summary, adjustment for variables, and items of methodological quality. The study quality was evaluated using a 9-point Newcastle–Ottawa Scale (NOS) (25). Studies with a score of 7 points or over were considered as high-quality.

### Data Synthesis and Analysis

All data were analyzed using STATA 12.0 (StataCorp, TX, USA). To facilitate meta-analysis, the OR and HR reported in the original studies were directly considered as approximately RR. Heterogeneity between studies was checked using the I^2^ statistic and Cochran's Q test. A random-effect model was utilized if there was evidence of significant heterogeneity (I^2^ statistic > 50% and/or *P* value < 0.1 of Cochran's Q test); otherwise, we used a fixed-effect model for analysis. Subgroup analyses were performed according to study design, ACS subtypes, sample sizes, and length of follow-up. In addition, we conducted a leave-out-one study sensitivity analysis to examine the robustness of the original pooling risk estimate and sources of the heterogeneity. Publication bias was investigated by visual inspection of funnel plot if more than 10 studies were included in the analysis.

## Results

### Search Results and Study Characteristics

**Figure 1:**
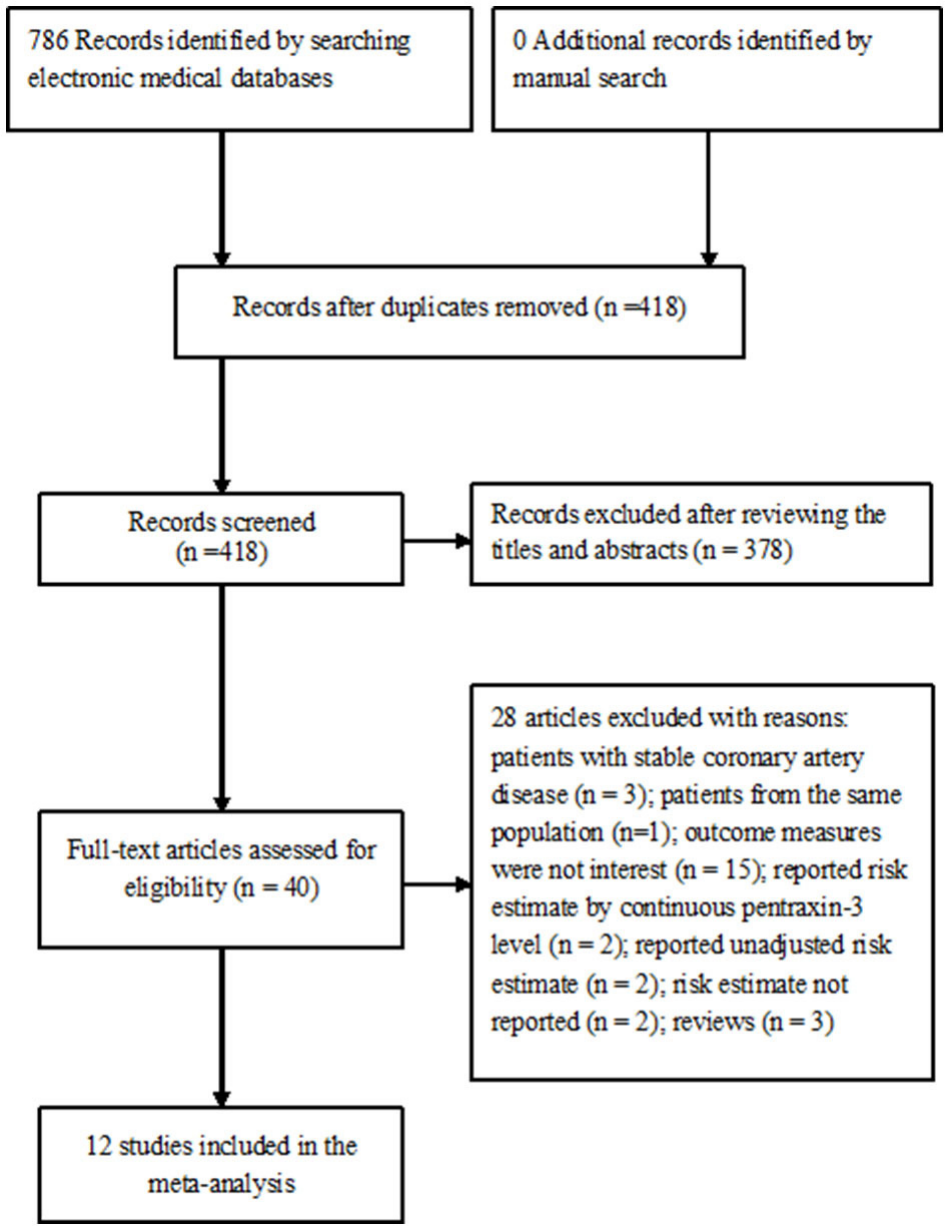
Flow diagram showing study selection process.

Figure 1 summarizes the process of study selection. A total of 786 potentially relevant records were identified by searching the electronic medical databases. After removing duplicated records and reviewing the titles and abstracts, 40 articles were retrieved for full-text evaluation. Of which, 28 articles were further removed after applying the predefined inclusion criteria. Thus, 12 studies (12–17, 21, 22, 26–29) were ultimately included in this meta-analysis.

Table 1 describes the baseline characteristics of the included studies. Two studies (13, 22) adopted the retrospective design and others were prospective studies. A total of 8,775 patients with ACS were identified, with sample sizes ranging from 79 to 5,154. The percentage of male gender ranged from 41.7 to 85.7%. Follow-up duration varied from 30 days to 84 months. According to the NOS criteria, the included studies were grouped as moderate to high quality (6–8 NOS points).

**Table 1:** Characteristics of the included studies.

**References**	**Region**	**Study design**	**Patients**	**Sample size (%men)**	**Age (years)**	**Definition of cardiac events**	**PTX 3 cutoff value (ng/ml)**	**Outcomes HR/RR (95% CI)**	**Follow-up (months)**	**Maximal adjusted variables**	**Total NOS**
Latini et al. (12)	Italy	P	AMI	724 (69.1)	Not provided	–	>10.7 vs. ≤ 5.5	Total death 3.55 (1.43–8.83)	3.0	Age, sex, smoking, hypertension, DM, Killip class, heart rate, SBP, anterior MI, creatine kinase	7
Guo et al. (13)	China	R	NSTEMI	525 (63.5)	57.7 ± 9.3	Recurrent MI, unstable angina pectoris, TVR	≥3.0 vs. <3.0	Cardiac events 1.36 (1.09–1.68); CV death 1.59 (1.18–2.15)	1.0	Age, gender, BMI, DM, SBP, DBP, LVEF hypertension, hyperlipidemia, smoker, heart rate, hsCRP, cTnT, NT-proBNP	7
Akgul et al. (14)	Turkey	P	STEMI	499 (79.6)	55.5 ± 12.3	–	≥3.2 vs. <3.2	Total death 2.3 (1.2–4.9)	24	Age, gender, DM, hypertension, Killip class, unsuccessful procedure, LVEF, anemia, creatinine, peak troponin	8
Mjelva et al. (21)	Norway	P	Suspected ACS	871 (61.3)	69.5 ± 14.4	Cardiac death and recurrent non-fatal cardiac disease	>9.5 vs. <3.5	Total death 1.62 (1.11–2.37); Cardiac events 1.39 (0.83–2.33)^#^	84	Age, sex, smoking, hypertension, eGFR, DM, NYHA, CAD, hypercholesterolemia, HF, cTnT, BNP, hsCRP	8
Altay et al. (15)	Turkey	P	AMI	140 (72.9)	59.7 ± 12.3	–	≥4.27 vs. ≤ 1.63	CV death 2.18 (1.43–3.32)	60	LVEF, hsCRP, NT-proBNP Global Registry of Acute Coronary Events score, TIMI score	8
Qiu et al. (16)	China	P	STEMI	84 (75)	55.8 ± 13.3	Non-fatal MI or HF, cardiac death	≥6.90 vs. <2.53	Cardiac events 3.64 (1.34–7.59)	3.0	Age, gender, cTnI, NT-proBNP	6
Chen (17)	China	P	ACS	120 (41.7)	58.9 ± 12.5	Arrhythmia MI, HF, UAP,	High vs. low	Cardiac events 3.12 (1.10–8.84)	6.0	Age, smoking, hypertension, DM, hsCRP, chemokine 16	7
Ljuca et al. (26)	Tuzla	P	STEMI	97 (73.2)	67.1 ± 7.6	Cardiac death, non-fatal MI, TVR	≥5.04 vs. <5.04	Cardiac events 2.40 (1.39–4.29)	24	Age, hypertension, hyperlipidemia, LVEF, DM, smoking, cTnI, hsCRP, interleukin-6, interleukin-10, Killip class	7
Dharma et al. (28)	Indonesia	P	STEMI	335 (85.7)	47–63	Cardiac death, non-fatal MI, TVR	>4.38 vs. <4.38	Total death 11.8 (1.52–92.3)	1.0	Age, sex, DM, hypertension, anterior MI, leukocyte, creatinine, random blood glucose	8
Zagidullin et al. (29)	Russia	P	STEMI	147 (80.3)	60.9 ± 12.1	–	>169 vs. ≤ 169	CV death 5.26 (2.23–12.4)	24	Age, gender, cTnI, LVEF	7
Kontny et al. (22)	Norway	R	ACS	5,154 (68.9)	52–73	CV death, spontaneous MI	>3.0 vs. <1.2	Cardiac events 1.30 (0.92–1.83) CV death 1.80 (1.03–3.15)	12	Age, gender, BMI, DM, CKD, hypertension, smoking, ACS type, history of HF, MI, PCI, CABG, stroke or PAD, leukocytes, hsCRP, interleukin-6, cystatin C	8
Jiang (27)	China	P	ACS	79 (51.9)	63.7 ± 8.27	Cardiac death and ACS readmission	>0.89 vs. ≤ 0.89	Cardiac events 3.02 (1.03–8.80)	3.0	Hypertension, DM, lipids, cTnI, hsCRP, C1q/tumor necrosis factor-related protein 9	7

*PTX 3, pentraxin 3; HR, hazard ratio; RR, risk ratio; P, prospective; R, retrospective; CV, cardiovascular; MI, myocardial infarction; AMI, acute MI; ACS, acute coronary syndrome; STEMI, ST segment elevated myocardial infarction; NSTEMI, non-STEMI; BMI, body mass index; DM, diabetes mellitus; SBP, systolic blood pressure; CKD, chronic kidney disease; hsCRP, high sensitivity CRP; TIMI, Thrombolysis in Myocardial Infarction; cTn, cardiac troponin; NT-proBNP, N-terminal pro-B-type natriuretic peptide; LVEF, left ventricular ejection fraction; eGFR, estimated glomerular filtration rate; PCI, percutaneous coronary intervention; CABG, coronary artery by-pass grafting; PAD, peripheral artery disease; TVR, target vessel revascularization; CAD, coronary artery disease; HF, heart failure; NOS, Newcastle-Ottawa Scale*.

# *Data from Mjelva et al. (21)*.

### Cardiac Events

**Figure 2:**
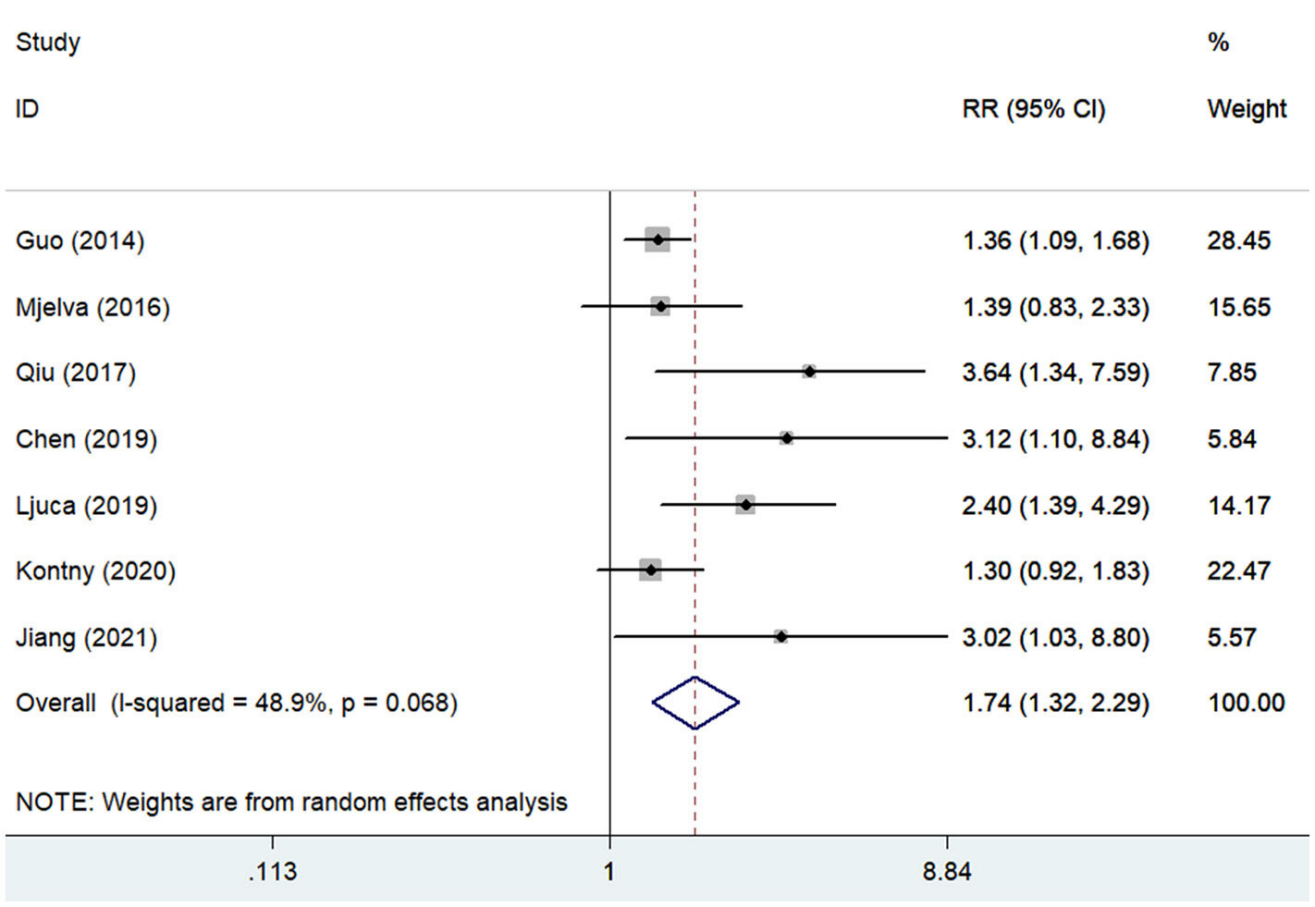
Forest plots showing the pooled multivariate-adjusted RR and 95% CI of cardiac events for the highest pentraxin-3 level vs. the lowest pentraxin-3 level.

Seven studies (13, 16, 17, 21, 22, 26, 27) reported the association of pentraxin-3 level with cardiac events. A random-effect meta-analysis indicated that elevated pentraxin-3 level was associated with an increased risk of cardiac events (RR 1.74; 95% CI 1.32–2.29; I^2^ = 48.9%; *P* = 0.068; Figure 2). Sensitivity analysis did not significantly alter the original predictive significance (data not shown). Subgroup analysis (Table 2) showed that the value of elevated pentraxin-3 in predicting cardiac events was stronger in STEMI patients (RR 2.72) than all patients with ACS (RR 1.59).

**Table 2:** Results of subgroup analyses on cardiac events.

**Subgroup**	**Number of studies**	**Pooled risk ratios**	**95% confidence intervals**	**Heterogeneity between studies**
**Study design**				
Prospective	5	2.26	1.55–3.29	*P* = 0.269; *I*^2^ = 22.8%
Retrospective	2	1.34	1.12–1.61	*P* = 0.828; *I*^2^ = 0.0%
**Subtypes of ACS**				
All ACS	4	1.59	1.10–2.29	*P* = 0.234; *I*^2^ = 29.7%
STEMI	2	2.72	1.69–4.36	*P* = 0.430; *I*^2^ = 0.0%
**Sample sizes**				
≥500	3	1.35	1.13–1.60	*P* = 0.969; *I*^2^ = 0.0%
<500	4	2.81	1.89–4.19	*P* = 0.873; *I*^2^ = 0.0%
**Length of follow-up**				
>6 months	3	1.55	1.09–2.21	*P* = 0.181; *I*^2^ = 41.5%
≤ 6 months	4	2.32	1.24–4.31	*P* = 0.040; *I*^2^ = 63.9%

*ACS, acute coronary syndrome; STEMI, ST segment elevation myocardial infarction*.

### All-Cause Mortality

**Figure 3:**
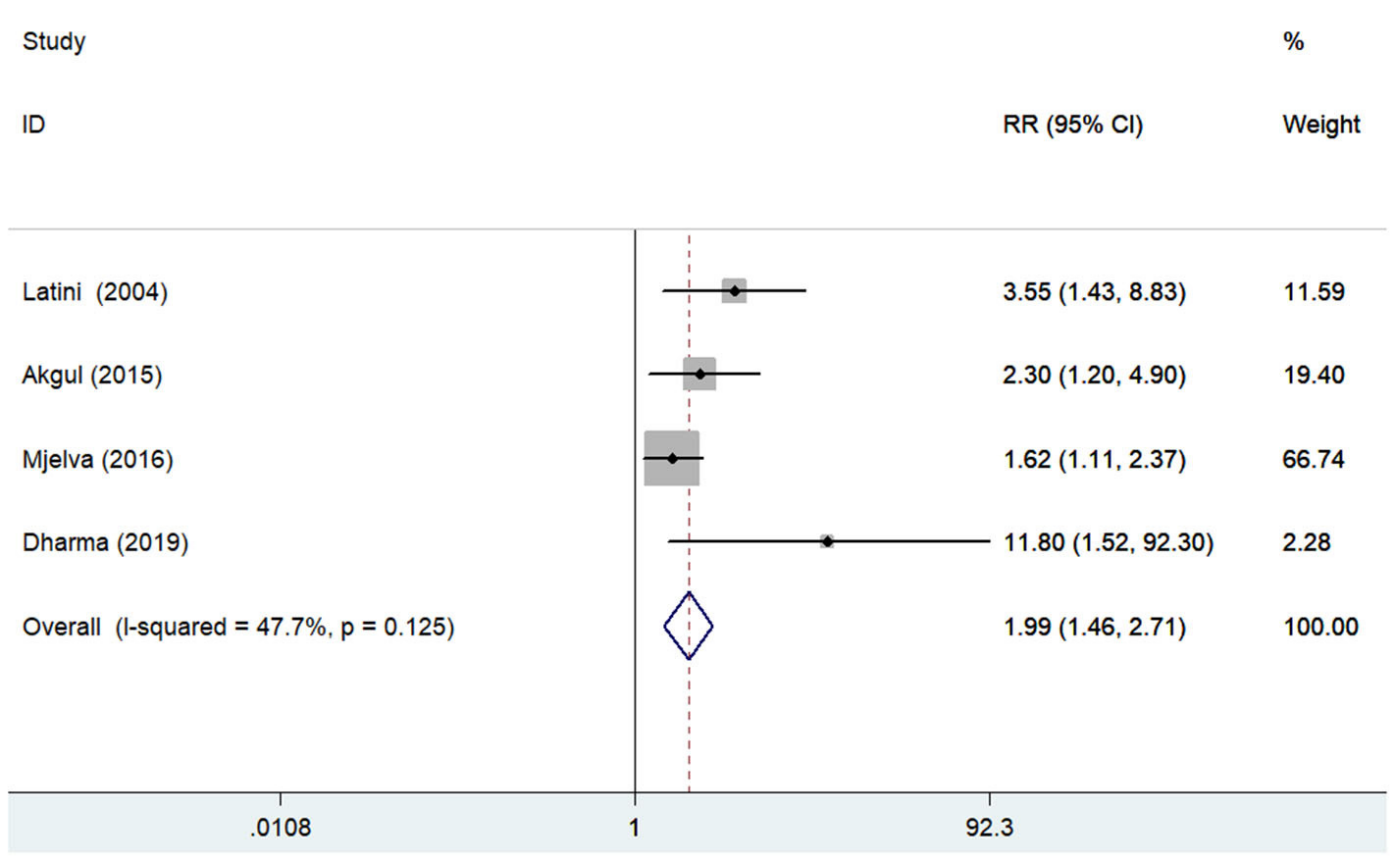
Forest plots showing the pooled multivariate-adjusted RR and 95% CI of all-cause mortality for the highest pentraxin-3 level vs. the lowest pentraxin-3 level.

Four studies (12, 14, 21, 28) reported the association between pentraxin-3 level and all-cause mortality. A fixed-effect meta-analysis showed that elevated pentraxin-3 level was associated with higher risk of all-cause mortality (RR 1.99; 95% CI 1.46–2.71; I^2^ = 47.7%; *P* = 0.125; Figure 3). Sensitivity analysis did not markedly change the originally predictive significance (data not shown).

### Cardiovascular Mortality

**Figure 4:**
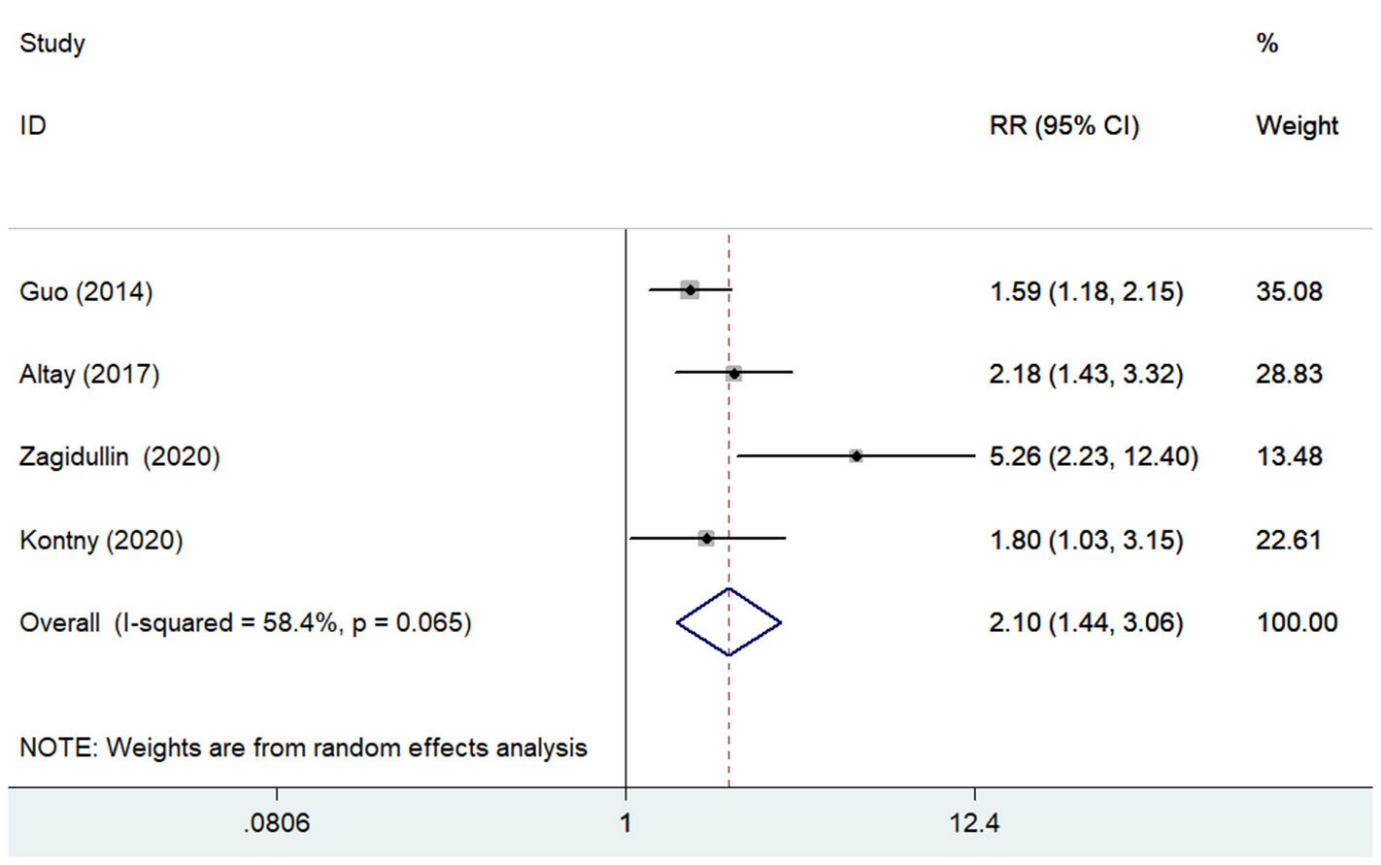
Forest plots showing the pooled multivariate-adjusted RR and 95% CI of cardiovascular mortality for the highest pentraxin-3 level vs. the lowest pentraxin-3 level.

Four studies (13, 15, 22, 29) reported the association of pentraxin-3 level with cardiovascular mortality. A random-effect meta-analysis showed that elevated pentraxin-3 level was associated with a higher risk of cardiovascular mortality (RR 2.10; 95% CI 1.44–3.06; I^2^ = 58.4%; *P* = 0.065; Figure 4). Sensitivity analysis did not significantly change the original predictive significance (data not shown).

### Publication Bias

Because of less than recommended an arbitrary number of 10 studies, we did not construct the funnel plots to examine the likelihood of publication bias (30).

## Discussion

The main finding of the current meta-analysis indicates that elevated blood level of pentraxin-3 is significantly associated with a higher risk of cardiac events, cardiovascular mortality, and all-cause mortality in patients with ACS, even after adjustment for confounders. ACS patients with the highest pentraxin-3 level conferred ~74%, 1.99-fold, and 2.1-fold higher risk of cardiac events, all-cause mortality, and cardiovascular mortality, respectively.

In addition to categorical data analysis of pentraxin-3 level, each ten-fold increase in pentraxin-3 level was associated with a 3.86-fold higher risk of cardiac events in patients with NSTEMI and unstable angina (19). Moreover, per 50% increase in acute phase pentraxin-3 level was associated with a 7% higher risk of cardiovascular mortality after adjustment for confounders in patients with ACS (11). These findings further supported the predictive role of elevated pentraxin-3 level in patients with ACS. However, the controversial result remained using continuous variable analysis of pentraxin-3 in patients with non-ST-elevation ACS (20).

Acute coronary syndrome (ACS) includes heterogeneous patient populations. Our subgroup analysis showed that the value of elevated pentraxin-3 in predicting cardiac events appeared to be stronger in STEMI patients compared with all ACS patients. This result may be correlated with a higher median pentraxin-3 level in STEMI than in NSTEMI patients (31). Pentraxin-3 level was significantly correlated with infarct size (32) and thrombus burden (28). When we grouped the studies by the length of follow-up, the value of pentraxin-3 in predicting cardiac events was evident in studies with short-term follow-up than those with relatively long-term follow-up. This finding suggested that the pentraxin-3 level may provide important information for the early risk stratification of ACS patients. Notably, the results of subgroup analysis should be interpreted with caution due to the limited number of studies analyzed.

Pentraxin-3 is an early biomarker of local inflammation in the vasculature (33). Blood pentraxin-3 level positively correlated with cardiac troponin I, creatine kinase-MB, and high-sensitivity CRP (15, 27). Elevated pentraxin-3 level may reflect an acute inflammatory response induced by myocardial injury. Moreover, the pentraxin-3 level was correlated with plaque vulnerability (34). Both the degree of inflammation and plaque vulnerability contribute to the adverse clinical events. Pentraxin-3 and CRP may represent different inflammatory processes of atherosclerosis. The increased blood level of the pentraxin protein family member CRP was also associated with a higher long-term risk of recurrent cardiovascular events and mortality in patients with ACS (35). An interesting issue is whether the predictive value of pentraxin-3 is superior to CRP. Considering pentraxin-3 level peaked earlier than CRP (22, 36), therefore it may be a better prognostic marker than CRP in acute phase ACS (19, 21, 26). Also, the pentraxin-3 level was more closely associated with the angiographic complexity and severity of coronary disease compared with CRP (37).

This meta-analysis has potential implications for clinical practice. Detection of blood pentraxin-3 level has the potential to identify high-risk ACS patients. ACS patients with elevated pentraxin-3 level should be received more closely monitor and active evidence-based therapies. Correspondingly, ACS patients with a high level of pentraxin-3 may potentially benefit from anti-inflammatory therapies. However, future well-designed clinical trials are necessary to support this hypothesis.

There were several potential limitations in our meta-analysis. First, a single measurement of the blood level of pentraxin-3 rather than a dynamic monitor may have resulted in selection bias. Second, different cut-off points of pentraxin-3 elevation used across studies prevent clinicians to identify those in need of closely monitor and active treatment. Future studies should further determine the optimal cut-off value for pentraxin-3 elevation. Third, we failed to analyze the predictive role of pentraxin-3 by continuous data because of the small number of such studies. Fourth, the majority of study outcomes were limited by considerable and at least moderate heterogeneity (~50% or more), which may be partly interpreted by the different cut-off points of pentraxin-3 elevation, the definition of outcomes, subtypes of ACS, or length of follow-up. Finally, we pooled the studies reporting different risk estimates (RR, OR, and HR) and approximated OR and HR to RR. This approach may have led to a slight overestimation of actual predictive value.

## Conclusions

This meta-analysis suggests that the blood level of pentraxin-3 as an inflammatory marker possibly independently predicts cardiac events, cardiovascular mortality, and all-cause mortality in patients with ACS. Detection of acute-phase pentraxin-3 level can provide important information for early risk stratification of ACS.

## Data Availability Statement

The original contributions presented in the study are included in the article/supplementary material, further inquiries can be directed to the corresponding author.

## Author Contributions

DG: study conception, design, and revised the article critically for important intellectual content. YF and RH: acquisition of data, analysis, and interpretation of data. YF and CM: statistical analysis. YF drafted the article. All authors read the final approval of the version to be published.

## Funding

This work is supported by (1) the Jiangsu Innovative Team Leading Talent Fund (CXTDC2016006, QNRC2016446); (2) the Zhenjiang Key Research and Development Fund (SH2021038), and (3) the Suqian Science and Technology Support Project Fund (K201907).

## Conflict of Interest

The authors declare that the research was conducted in the absence of any commercial or financial relationships that could be construed as a potential conflict of interest.

## Publisher's Note

All claims expressed in this article are solely those of the authors and do not necessarily represent those of their affiliated organizations, or those of the publisher, the editors and the reviewers. Any product that may be evaluated in this article, or claim that may be made by its manufacturer, is not guaranteed or endorsed by the publisher.
